# Structural and Performance Studies of Lanthanum–Nitrogen Co-Doped Titanium Dioxide Thin Films Under UV Aging

**DOI:** 10.3390/mi16080842

**Published:** 2025-07-23

**Authors:** Pengcheng Cao, Li Zhang, Yanbo Yuan

**Affiliations:** College of Science, Inner Mongolia University of Technology, Hohhot 010051, China; pengcheng0831@163.com (P.C.); yyb2624095231@gmail.com (Y.Y.)

**Keywords:** lanthanum–nitrogen co-doped titanium dioxide (La-N-TiO_2_) thin films, ion beam assisted deposition (IBAD), ultraviolet (UV) aging, self-cleaning, stability

## Abstract

In this study, lanthanum–nitrogen co-doped titanium dioxide (La-N-TiO_2_) thin films were fabricated using Ion Beam Assisted Deposition (IBAD) and subjected to accelerated ultraviolet (UV) aging experiments to systematically investigate the impact of co-doping on the films’ resistance to UV aging. X-ray diffraction (XRD) analysis revealed that La-N co-doping inhibits the phase transition from anatase to rutile, significantly enhancing the phase stability of the films. Scanning electron microscopy (SEM) and atomic force microscopy (AFM) characterizations indicated that co-doping increased the density and surface uniformity of the films, thereby delaying the expansion of cracks and increase in roughness induced by UV exposure. Energy-dispersive X-ray spectroscopy (EDS) results confirmed the successful incorporation of La and N into the TiO_2_ lattice, enhancing the chemical stability of the films. Contact angle tests demonstrated that La-N co-doping markedly improved the hydrophobicity of the films, inhibiting the rapid decay of hydrophilicity during UV aging. After three years of UV aging, the co-doped films maintained high structural integrity and photocatalytic performance, exhibiting excellent resistance to UV aging. These findings offer new insights into the long-term stability of photovoltaic self-cleaning materials.

## 1. Introduction

In the context of escalating energy crises and environmental pollution, the development of efficient, sustainable new energy sources and environmental materials has emerged as a focal point in global scientific research [1,2]. The photovoltaic industry, a crucial component of clean energy solutions, plays an essential role in the long-term stable operation necessary for achieving sustainable energy development. However, photovoltaic panels in outdoor environments are frequently affected by the accumulation of dust, dirt, and environmental pollutants, which reduce their photoelectric conversion efficiency [3]. Particularly under the combined effects of sand erosion and ultraviolet (UV) aging [4], the long-term stability of self-cleaning thin films becomes a critical factor influencing the continuous high-efficiency operation of photovoltaic panels [5]. Traditional self-cleaning coatings often exhibit diminished hydrophilicity and damaged surface structures after a period of use, leading to decreased light absorption and weakened self-cleaning performance. Hence, enhancing the stability of self-cleaning thin films under conditions of sand erosion and UV aging has become a pressing scientific issue.

Among various self-cleaning materials, titanium dioxide (TiO_2_) thin films stand out due to their exceptional photocatalytic performance and superhydrophilic properties [6]. These films show vast potential in applications such as self-cleaning coatings, architectural glass, photovoltaic modules, and transportation infrastructure. Yet, the practical application of TiO_2_ thin films faces certain limitations, including (1) a narrow UV light response range, capable only of absorbing UV light with wavelengths below 387 nm, which results in low visible light utilization [7]; (2) a high rate of electron–hole pair recombination, limiting the efficiency of photogenerated carrier utilization and constraining enhancements in photocatalytic performance [8]; and (3) insufficient long-term stability under intense UV conditions, leading to photoinitiated aging, phase transformation, optical property degradation, and surface structure deterioration [9]. These factors significantly restrict the long-term stable application of TiO_2_ thin films in outdoor environments. Consequently, doping modification has become an effective method to improve the photocatalytic performance and UV aging stability of TiO_2_ thin films.

Element doping can modulate the electronic structure of TiO_2_, introduce energy level defects, and inhibit electron–hole recombination, thereby enhancing its photocatalytic activity and stability [10]. Single-element doping with either metals or non-metals can, to a certain extent, improve the performance of TiO_2_. For instance, Yang et al. (2025) discovered that doping with La^3+^ introduces oxygen vacancies in TiO_2_ thin films, effectively enhancing the absorption of light and the mobility of photogenerated carriers, thereby increasing photocatalytic efficiency [11]. Zhu et al. (2018) demonstrated that La^3+^ doping significantly suppresses the phase transformation from anatase to rutile in TiO_2_ thin films, thus enhancing the films’ humidity stability [12]. Additionally, Chakraborty et al. (2023) showed that N doping introduces intermediate energy levels within the band gap of TiO_2_, expanding the light response range and enhancing light absorption in the visible spectrum, which in turn boosts photocatalytic activity [13].

The synergistic effect of La and N co-doping in TiO_2_ arises from their complementary roles in modifying the crystal and electronic structure. La^3+^ ions, when substituted into the Ti^4+^ lattice sites due to their larger ionic radius, induce lattice distortion and generate oxygen vacancies (V) to maintain charge neutrality. These oxygen vacancies act as shallow traps that facilitate charge separation and enhance carrier mobility. Simultaneously, N atoms replace lattice oxygen (O^2−^), introducing localized states slightly above the valence band, which results in bandgap narrowing and improved visible light absorption.

More importantly, La doping promotes the formation of oxygen vacancies, which in turn provide favorable conditions for stabilizing N dopants in the lattice. This mutual facilitation between La and N enhances the formation energy of co-doped configurations and suppresses electron–hole recombination by reducing trap-assisted recombination centers. Density Functional Theory (DFT) calculations confirm that La-N co-doping results in a new hybrid electronic structure with reduced recombination probability and improved charge carrier separation, thereby synergistically boosting the photocatalytic and aging-resistance performance of TiO_2_ films.

Although single doping with either La or N can improve the optical and structural properties of TiO_2_, each has its limitations. Doping with La^3+^ enhances stability but has a limited effect on extending the light response range [14], whereas N doping, despite enhancing visible light absorption, can lead to increased carrier recombination, reducing photocatalytic efficiency. Consequently, we propose a strategy of lanthanum–nitrogen (La-N) co-doping to overcome the limitations of single-element doping and further enhance the photocatalytic activity and UV aging resistance of TiO_2_ thin films. La-N co-doping not only facilitates the introduction of oxygen vacancies through La^3+^, providing favorable conditions for N doping, but also synergistically improves the separation efficiency of photogenerated carriers, effectively suppressing electron–hole recombination, thus significantly enhancing the photocatalytic activity and UV aging stability of the TiO_2_ thin films [15].

UV aging is a critical factor influencing the long-term performance of TiO_2_ thin films. Under prolonged exposure to UV radiation, TiO_2_ thin films undergo both physical and chemical alterations, such as phase transitions, increased surface roughness, changes in the band gap, and a decline in photocatalytic performance [16]. Studies have shown that under intense light exposure, the anatase phase of pure TiO_2_ thin films gradually transitions to the rutile phase, which exhibits significantly lower photocatalytic activity compared to the anatase phase. For instance, Zhao et al. (2025) discovered that pure TiO_2_ nanoparticles, when subjected to strong UV radiation, undergo a phase transition from anatase to rutile, resulting in a substantial decrease in photocatalytic performance [17]. Additionally, UV irradiation can induce a blue shift in the band gap of TiO_2_ thin films, reducing their light absorption capacity and thereby diminishing their photocatalytic performance. Pan Yongqiang et al. (2013) utilized electron beam evaporation to fabricate TiO_2_ thin films and studied the changes in their optical properties following UV light irradiation, noting a decrease in both refractive index and transmittance over time, along with a significant reduction in photocatalytic activity [18].

Recent studies have explored La-N co-doped TiO_2_ thin films to improve photocatalytic activity and visible light responsiveness. However, most of these studies primarily focus on the photocatalytic performance under laboratory conditions, with limited attention paid to the long-term structural and optical stability of co-doped TiO_2_ thin films under intense UV exposure, which is critical for real-world outdoor applications, such as photovoltaic panel coatings. Additionally, conventional fabrication techniques used in previous work (e.g., sol–gel, hydrothermal synthesis) often result in films with poor mechanical integrity and weak adhesion, limiting their durability under environmental stressors.

To address these limitations, this study employs an ion beam-assisted deposition (IBAD) technique to prepare La-N co-doped TiO_2_ thin films with enhanced mechanical robustness. More importantly, this work aims to fill the research gap by systematically investigating the UV-aging-induced degradation mechanisms of La-N-TiO_2_ films through a combination of structural, morphological, and optical characterizations. These insights will help to better understand how co-doping influences long-term film performance and stability in harsh environments.

## 2. Materials and Methods

### 2.1. Preparation of Thin Films

**Figure 1:**
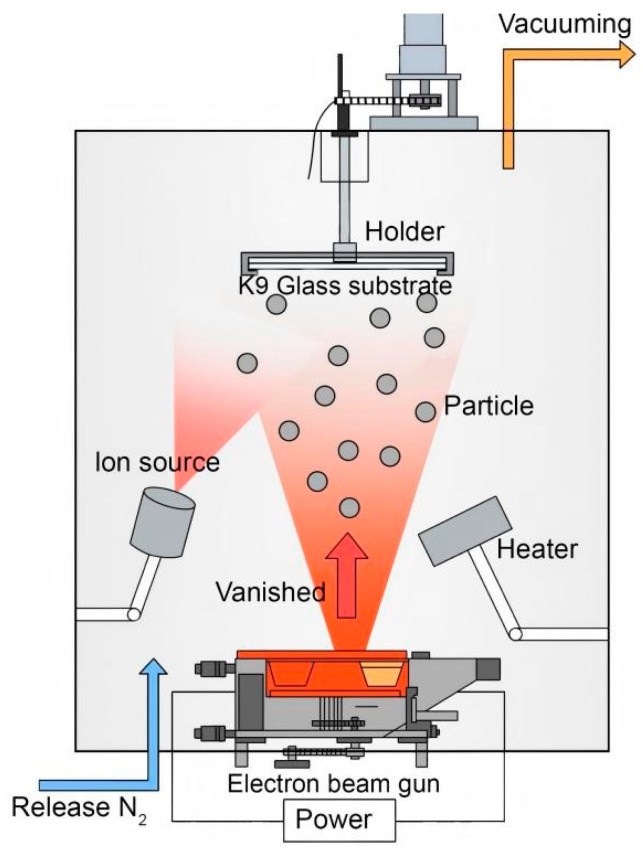
Schematic diagram of IBAD coating process.

The preparation of thin films was conducted using IBAD as schematically illustrated in Figure 1. Prior to deposition, K9 glass substrates were ultrasonically cleaned using deionized water, ethanol (Fuyu Chemical, Tianjin, China), and acetone (Fuyu Chemical, Tianjin, China), followed by plasma cleaning to remove surface contaminants and enhance the adhesion of the thin films. Subsequently, in a high vacuum environment (5 × 10^−5^ Pa), high-purity nitrogen gas (10–20 sccm, 1.4–2.0 Pa) was introduced as a doping source. The target materials, TiO_2_ and La_2_O_3_ in a 97:3 mass ratio, were heated to 1600–1700 °C using electron beam evaporation to facilitate vaporization while maintaining a deposition rate of 0.1–0.5 nm/s. This ratio was selected based on previous studies, which found that La doping levels around 3% effectively enhance photocatalytic activity and film stability without introducing secondary phases [19]. 

**Figure 2:**
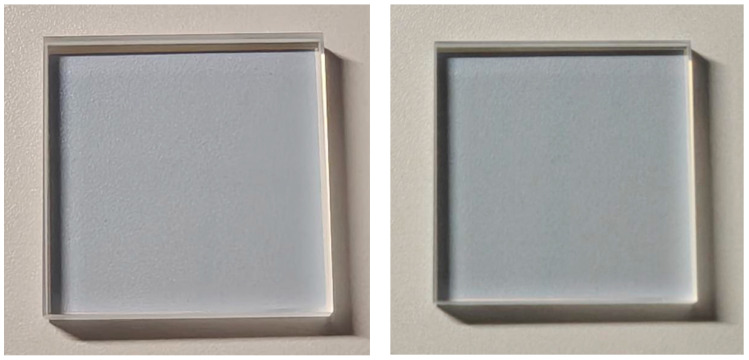
Photograph of La-N co-doped TiO_2_ thin films fabricated by ion beam-assisted deposition (IBAD). The films exhibit uniform thickness and good surface quality, providing a stable platform for subsequent structural and optical analyses, demonstrating their uniform coverage, transparency, and good adhesion to the substrate.

Concurrently, an ion source was activated using an argon atmosphere, and with ion bombardment at energies ranging from 100 to 300 eV, particle migration was promoted, thereby enhancing the density of the films, inhibiting grain growth, and improving doping uniformity. During the deposition of the thin films, the substrate temperature was maintained at 300 °C to obtain La-N co-doped TiO_2_ thin films with uniform thickness and good surface quality. Photographs of the resulting films are shown in Figure 2, illustrating their macroscopic appearance on K9 glass substrates. Post-deposition, the films were annealed in a nitrogen atmosphere at temperatures between 500 and 600 °C for two hours to further improve their crystallinity and photocatalytic performance.

### 2.2. Accelerated UV Aging of Thin Films

The accelerated UV aging experiments on the thin films were carried out using a UV aging test chamber, equipped with UVA-340 and UVB-313 fluorescent UV lamps, based on the principle of equivalent effect to simulate shortwave solar UV radiation [20]. The annual total solar radiation in regions with strong UV exposure is approximately 4858–6925 MJ/m^2^, with the median value being 5800 MJ/m^2^, of which about 5% is UV radiation, resulting in an annual UV radiation exposure of approximately 290 MJ/m^2^.

$$\begin{array}{ll} Simulation\;time & =\frac{Annual\;UV\;radiation}{Laboratory\;UV\;intensity}\\ & =\frac{290\times 10^{6}÷3600}{1500}=53\;h \end{array}$$

To achieve accelerated aging, the UV radiation was converted into the power used by the UV aging test chamber (in kW·h). Under an irradiance of 1500 W/m^2^, theoretically, 53 h are required to simulate one year’s worth of UV exposure. However, considering the increase in temperature to approximately 60 °C during the experiments, which can accelerate the aging of the films, the duration was adjusted to 48 h to simulate the annual UV radiation. The experimental setup simulated UV exposure for 0, 1, 2, 3, and 5 years, as shown in Table 1.

In this study, the total UV irradiation dose was selected to simulate the equivalent of five years of natural outdoor exposure. This equivalence was based on ISO 4892-2 standards [21] and the related literature, which estimate that materials exposed outdoors typically receive an annual UV dose of approximately 200 MJ/m^2^ under mid-latitude sunlight conditions (e.g., in regions such as southern Europe or central China). Accordingly, a five-year exposure corresponds to a cumulative UV dose of around 1000 MJ/m^2^.

To accelerate the aging process, the UV chamber was operated at elevated irradiance levels (e.g., 1.5–2 times natural sunlight), which allows for achieving the equivalent five-year dose within a manageable test period while maintaining physical relevance.

The thin film samples were affixed within the UV aging fixture and labeled prior to placement in the UV aging chamber for unilateral exposure. During the tests, the temperature of the aging chamber was maintained at 50 ± 2 °C, with continuous irradiation of the samples.

### 2.3. Characterization and Mechanical Testing of Thin Films

The phase structure of the samples was characterized using a Rigaku D/MAX-2500/PC X-ray diffractometer (Rigaku Corporation, Tokyo, Japan) (Cu target, λ = 1.5406 Å, 40 kV, 40 mA, scanning angle range 5–90°, scanning speed 4°/min, step size 0.02°). The surface roughness of the thin films was measured using a Bruker Dimension Icon AFM (Bruker, Ettlingen, Germany) in tapping mode, with a scanning area of 4 μm × 4 μm, lateral resolution of 0.2 nm and vertical resolution of 0.05 nm. The microstructure and elemental composition were examined using a Hitachi SU1080 SEM (Hitachi High-Tech, Tokyo, Japan) operating at 5 kV, with a working distance of 21 mm, magnification of 2000×, and equipped with an EDS system covering elements from boron (B) to uranium (U). A Bruker TS 77 Nano Scratch Tester (Bruker, Germany) was utilized to perform nano-scratch tests, analyzing the friction coefficient and film adhesion.

## 3. Results and Discussion

### 3.1. XRD Analysis

**Figure 3:**
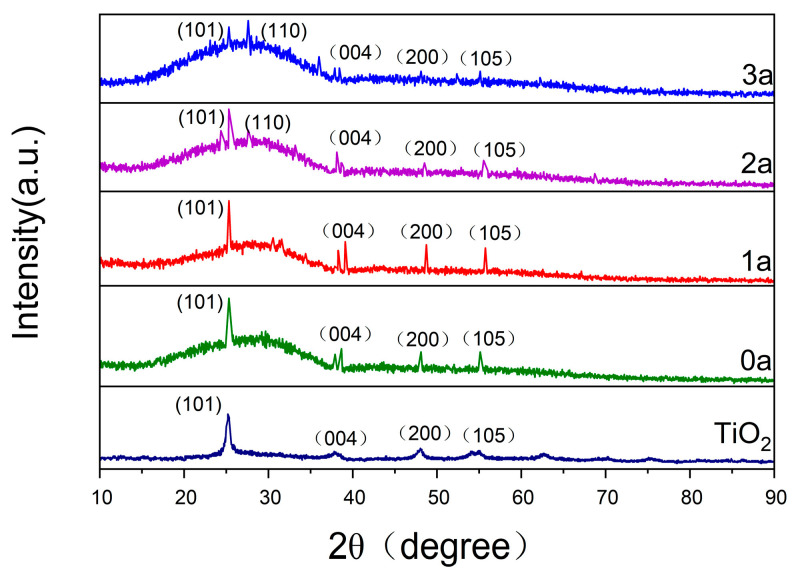
XRD patterns of La-N co-doped TiO_2_ thin films subjected to UV aging for 0, 1, 2, 3, and 5 years. The diffraction peaks remain sharp and well-defined over time, indicating that La-N co-doping effectively improves the crystallinity and inhibits the anatase-to-rutile phase transition during prolonged UV exposure.

Figure 3 illustrates the XRD patterns of La-N-TiO_2_ and TiO_2_ thin films after varying durations of UV aging (0, 1, 2, 3 years). The main diffraction peaks of TiO_2_ thin films are observed at 25.3° (101), 37.8° (004), 48.0° (200), and 53.9° (105), corresponding to the anatase TiO_2_ crystal structure [22]. The diffraction peaks of the unaged La-N-TiO_2_ thin films are more pronounced compared to those of the TiO_2_ thin films, indicating that La-N co-doping effectively enhances the crystallinity of the TiO_2_ films. In the early stages of aging (1 year), the peak intensities of the anatase phase increase, suggesting that UV light promotes the crystallization of some amorphous regions [23]. However, as aging progresses (2 and 3 years), the rutile phase gradually emerges and intensifies, while the proportion of the anatase phase decreases, indicating that prolonged UV exposure leads to the transformation of anatase to rutile. Compared to the TiO_2_ thin films, the La-N-TiO_2_ thin films exhibit a significantly slower rate of phase transformation, demonstrating the stabilizing effect of La at grain boundaries and the mitigating impact of nitrogen doping on lattice distortions. Compared to undoped TiO_2_ films, the La-N co-doped films exhibited slower phase transformation rates and better retention of anatase structure after 3 years of UV aging, confirming enhanced structural stability.

### 3.2. SEM and EDS Analysis

**Figure 4:**
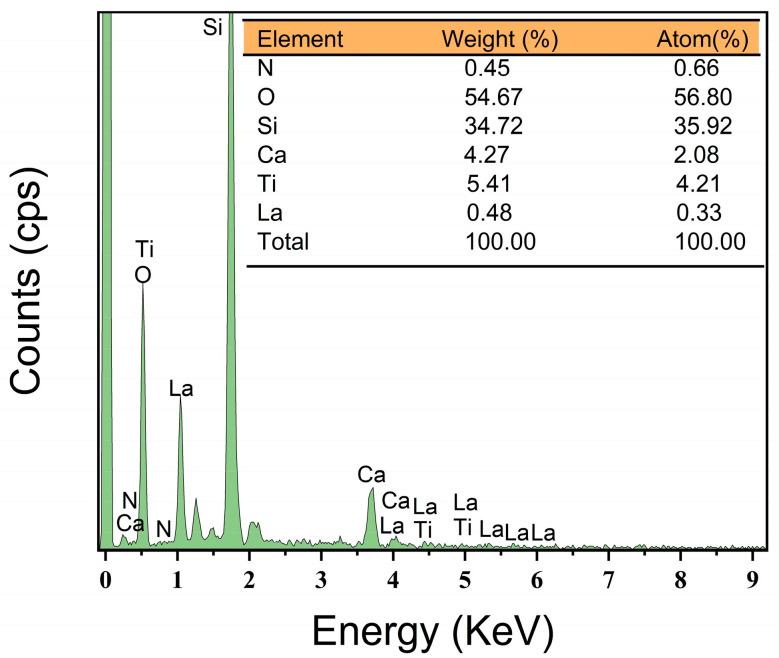
EDS spectrum of La-N co-doped TiO_2_ thin films. The presence of La and N confirms the successful incorporation of both dopants into the TiO_2_ matrix. Peaks corresponding to Ga and Si originate from the K9 glass substrate, rather than from the film itself.

Figure 4 presents the EDS spectrum of La-N-TiO_2_ thin films. The data indicate that in the unaged La-N-TiO_2_ thin films, the concentration of La is 0.33 atomic percent (at.%) and that of N is 0.66 at.%. Coupled with the XRD analysis presented earlier, these results demonstrate that the La-N co-doping effectively inhibits the transformation of crystalline grains towards the rutile phase. The substitution of some Ti^4+^ ions with La^3+^ ions leads to lattice expansion and the formation of microstrains, which in turn suppress grain growth. Additionally, the incorporation of nitrogen introduces Ti–N bonds, improving the distribution of electron clouds, retarding the recombination of photogenerated carriers, and maintaining the high crystallinity of the thin films [24,25,26].

**Figure 5:**
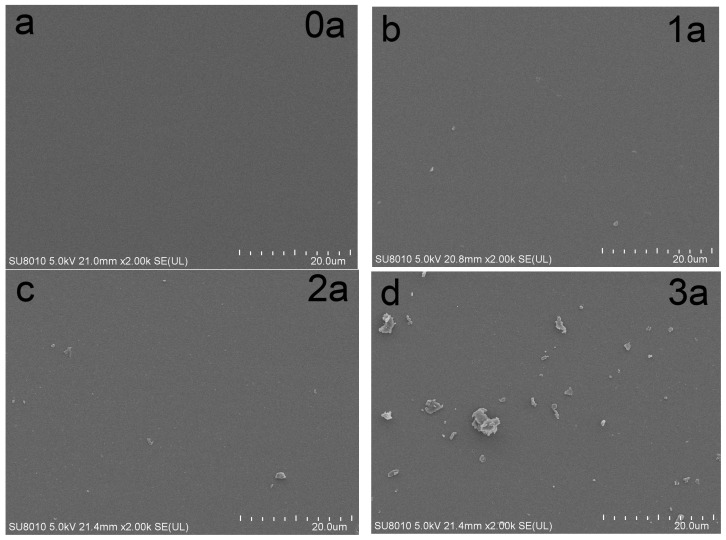
SEM images of La-N-TiO_2_ thin films at different aging times. The labels “0a”, “1a”, “2a”, and “3a” refer to films aged for 0, 1, 2, and 3 years, respectively, where “a” stands for “years”. The results show that La-N co-doping effectively inhibits the anatase-to-rutile phase transition under prolonged UV exposure, maintaining the anatase structure and indicating superior structural stability. (**a**) UV Aging 0 year; (**b**) UV Aging 1 year; (**c**) UV Aging 2 year; (**d**) UV Aging 3 year.

Figure 5 depicts SEM images of La-N-TiO_2_ thin films at various aging stages. Figure 5a reveals that La and N are successfully doped into the TiO_2_ lattice without forming impurity phases; the surface of the thin films is dense and devoid of evident cracks. This suggests that the La-N co-doping enhances the density of the TiO_2_ thin films, thereby inhibiting grain growth and the formation of cracks. Compared with undoped TiO_2_ films, which showed extensive crack propagation and surface fragmentation after prolonged UV exposure, the La-N co-doped films maintained a denser morphology with fewer and smaller cracks, as observed in the SEM images (not shown here for undoped films).

Figure 5b–d show that as the aging duration increases, the number of cracks and voids in the thin films also increases. The formation of these defects is primarily due to hydroxyl radicals generated by photocatalysis and thermal stress induced by UV radiation. Although La-N doping delays the propagation of cracks, it cannot entirely prevent their formation. The number and size of cracks tend to stabilize after 2–3 years of aging, and the grain boundary passivation effect of La significantly reduces the rate of crack propagation, thus endowing the thin films with superior resistance to UV aging.

While XPS was not conducted in this study, the EDS results (Figure 4) clearly confirm the successful incorporation of La and N into the TiO_2_ matrix. The presence of La and N, along with the observed changes in crystallinity and microstructure, indirectly supports the occurrence of lattice modification and defect formation due to co-doping. These effects are consistent with previous reports on La- and N-doped TiO_2_ thin films.

### 3.3. AFM Analysis

**Figure 6:**
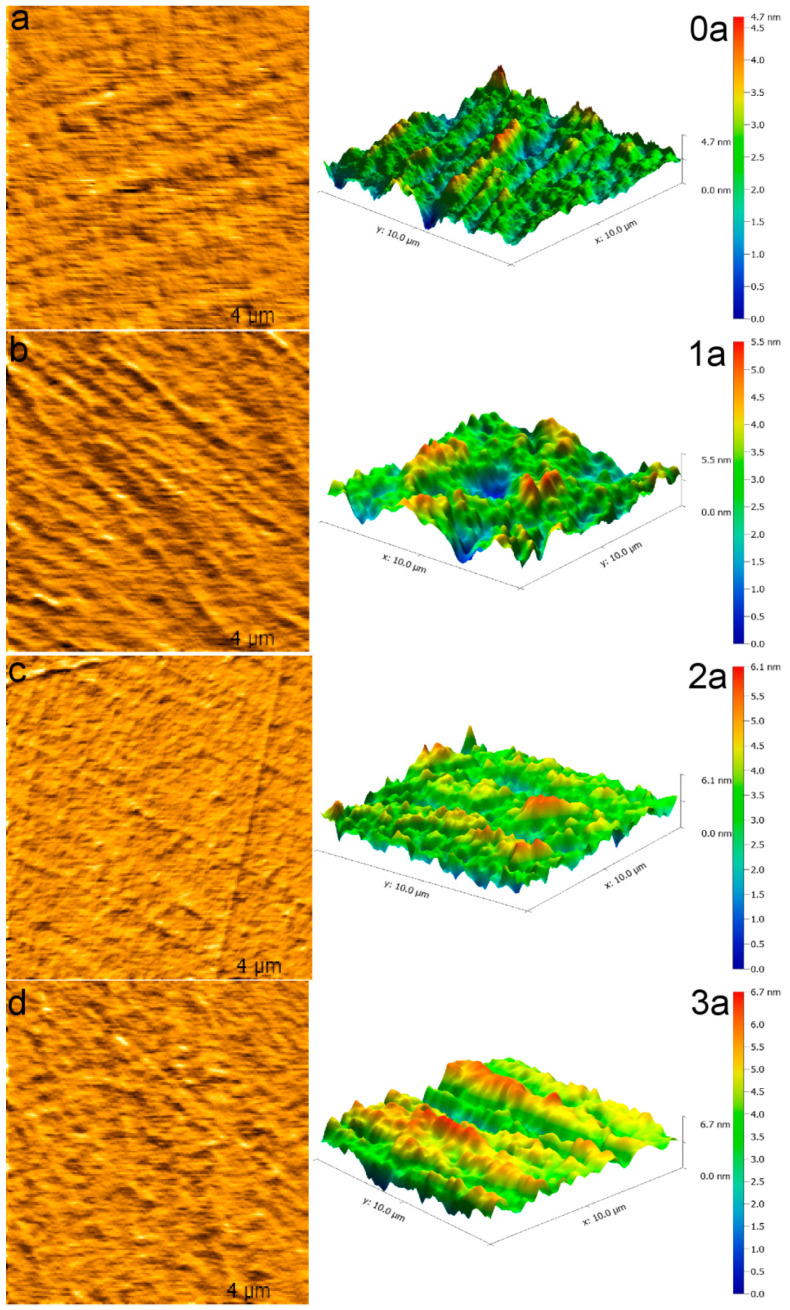
AFM images of La-N-TiO_2_ thin films at different aging stages in Table 2. Each data point represents the average of three repeated AFM scans. The observed variation was within ±5%, confirming good repeatability. (**a**) UV Aging 0 year; (**b**) UV Aging 1 year; (**c**) UV Aging 2 year; (**d**) UV Aging 3 year.

Figure 6 and Table 2 present the 2D and 3D topographies of the La-N-TiO_2_ thin films before and after aging as captured by AFM. Both SEM and AFM analyses confirm that the surface of the unaged thin films is dense and exhibits uniform grain sizes (average diameter of 2.244 nm) with a low roughness (Ra = 422.6 pm). Post exposure to UV light, the thin films’ surfaces progressively develop pores, cracks, and particle agglomeration. With aging for three years, the grain size increases to 4.288 nm, and both Ra and RMS escalate to 683.3 pm and 865.0 pm, respectively. Coupled with XRD and SEM analysis, the deterioration in surface morphology is closely associated with UV-induced grain growth, migration of oxygen vacancies, and surface etching. The co-doping of La-N significantly inhibits grain boundary migration and surface atom rearrangement, thereby effectively decelerating the increase in surface roughness. After three years of aging, the roughness only increased by approximately 62%, demonstrating exceptional UV aging resistance. In contrast, undoped TiO_2_ films typically exhibit roughness values exceeding 1000 pm under similar UV exposure, indicating that La-N co-doping effectively reduces surface degradation. This improvement can be attributed to inhibited grain boundary migration and enhanced structural cohesion.

### 3.4. Contact Angle Analysis

**Figure 7:**
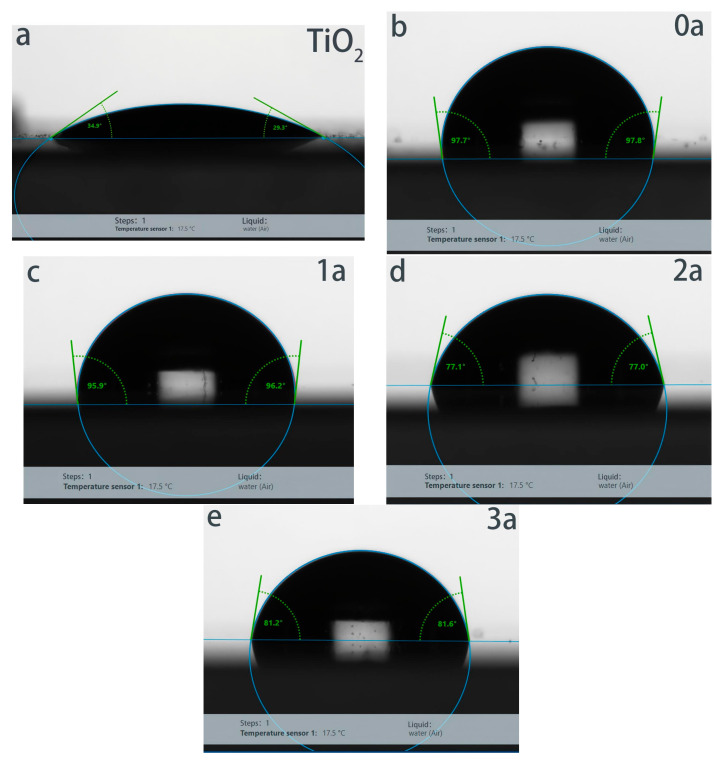
Contact angle measurements of La-N co-doped TiO_2_ thin films after different durations of UV aging (0–5 years). The initial contact angle of approximately 97° indicates strong hydrophobicity. Despite prolonged UV exposure, which typically promotes the formation of hydrophilic hydroxyl groups, the films maintain a relatively stable contact angle above 80°, demonstrating durable hydrophobic performance due to La-N co-doping. Error bars are omitted for clarity. Each data point represents the average of three measurements, with standard deviations less than 3°. (**a**) Pure titanium dioxide film; (**b**) UV Aging 0 year; (**c**) UV Aging 1 year; (**d**) UV Aging 2 year; (**e**) UV Aging 3 year.

Figure 7 illustrates the changes in the contact angles of La-N-TiO_2_ thin films during the process of UV aging, shifting gradually from an initial 98° to 96°, 77°, and finally 82°. This variation is intimately linked to the evolution of the film’s surface structure and chemical state [27]. Compared to pure TiO_2_ thin films (contact angles between 29° and 34°), La-N co-doping notably enhances the hydrophobicity and UV stability of the films. One year into aging, La-N co-doping effectively suppresses the recombination of photo-generated electrons and holes, reduces the rate of surface hydroxyl formation, and facilitates the formation of a La-O-Ti network, thereby enhancing the films’ resistance to UV degradation [28]. As a result, the contact angle only slightly decreases to 96°. After two years, although UV exposure triggers surface photocatalytic reactions that generate a substantial amount of hydrophilic hydroxyl groups (–OH), the La-N co-doping slows down the rate of hydrophilization, keeping the contact angle at 77°, significantly above that of pure TiO_2_ films. After three years, due to the re-adsorption of organic pollutants and dust from the air and the restructuring of the surface, some regions regain hydrophobicity, with the contact angle rebounding to 82°. Overall, La-N co-doping not only significantly boosts the initial hydrophobicity of the thin films but also prolongs the surface hydrophilization during long-term UV aging, thus maintaining superior waterproof performance and stability.

### 3.5. Raman Spectroscopy Analysis

**Figure 8:**
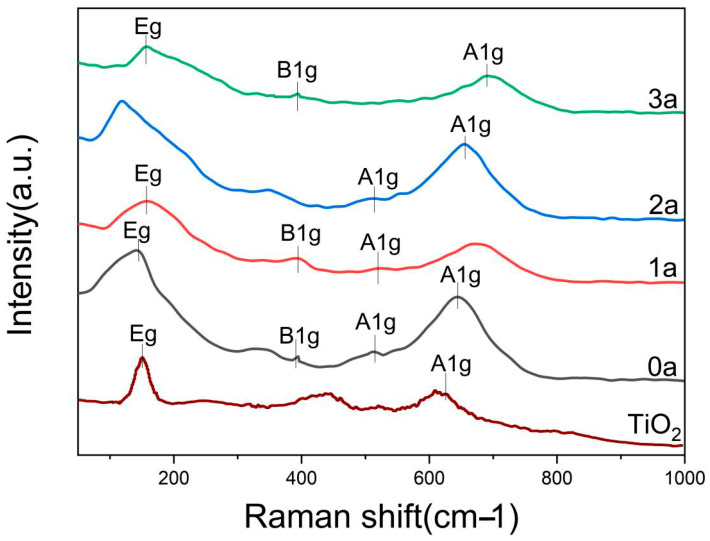
Contact angle spectra of La-N-TiO_2_ thin films at different aging times.

Figure 8 displays the Raman spectra of La-N-TiO_2_ thin films compared to pure TiO_2_ thin films over various durations of UV aging. The pure TiO_2_ films exhibit characteristic peaks of the anatase phase, including peaks at ≈151 cm^−1^ (Eg), ≈395 cm^−1^ (B1g), ≈517 cm^−1^, and ≈639 cm^−1^ (A1g), indicative of a well-defined crystalline structure with high orderliness. In the doped samples (0a, 1a, 2a, 3a), compared to pure TiO_2_, there is a general shift of the Eg peak from 149 cm^−1^ towards higher wavenumbers, culminating at 157 cm^−1^ for the 3a sample, with a concurrent increase in peak intensity. This suggests that the co-doping of La^3+^ and N^−^ introduces lattice stress, causing distortions in the Ti-O bonds and resulting in the shift of peak positions towards higher wavenumbers. However, at 2a aging, UV radiation leads to the crystallization of some amorphous regions and the migration of oxygen vacancies, causing a microstructural reconfiguration of the lattice, which temporarily shifts the Raman peaks towards lower wavenumbers. As aging progresses to 3a, increased lattice distortion provokes further shifts of the Eg and A1g peaks towards higher wavenumbers, and the A1g mode (≈665 cm^−1^) exhibits notable splitting and broadening. These changes reflect the combined effects of doping and UV aging on lattice distortion and structural degradation. The transient anomaly at 2a is closely associated with the local release of lattice stress and rearrangement of oxygen vacancies, while the overall shift towards higher wavenumbers reflects the persistent impact of doping-induced stress on the thin films’ lattice structure. The observed Raman peak shifts and broadening patterns further suggest that La and N dopants induce lattice stress and distortions. Although no XPS data are available, these spectroscopic features are commonly associated with changes in local bonding environments and defect concentrations, consistent with the effects expected from La–O–Ti and Ti–N bonding formation.

### 3.6. Mechanical Property Testing

**Figure 9:**
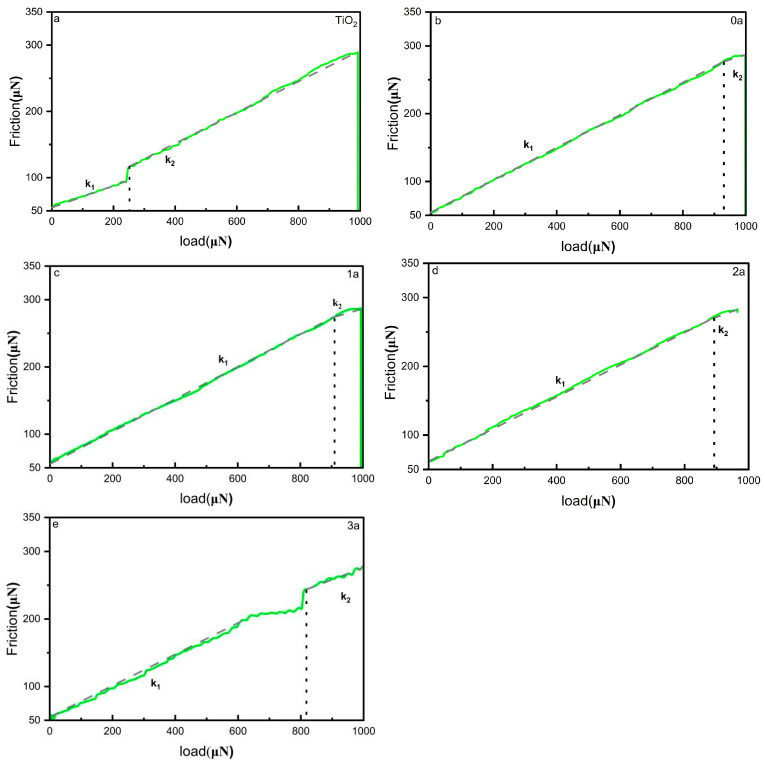
Nano-scratch test results for La-N co-doped TiO_2_ thin films, illustrating substrate adhesion strength under different UV aging conditions. The load–friction curves show that La-N co-doping significantly enhances interfacial bonding, as indicated by a higher critical load at the point of slope discontinuity (where k = f/n, the ratio of friction force to normal load). The results confirm that co-doping effectively mitigates mechanical degradation caused by prolonged UV exposure. Adhesion strength was measured in triplicate under identical conditions. The variation between tests was within ±5%, indicating good consistency. (**a**) Pure titanium dioxide film; (**b**) UV Aging 0 year; (**c**) UV Aging 1 year; (**d**) UV Aging 2 year; (**e**) UV Aging 3 year.

Data from the nano-scratch test, as shown in Figure 9, provide insights into the adhesion strength of the thin films to their substrates. Undoped TiO_2_ thin films exhibit an adhesion strength of 256 μN, indicating relatively low adhesion and weaker substrate–film bonding. After doping with La-N, the adhesion strength significantly increases to 936 μN, demonstrating that doping enhances the interfacial bonding capacity between the film and the substrate, improving adhesion. Following various durations of UV aging, the adhesion strength of the doped films gradually decreases to 895 μN, 860 μN, and 816 μN. However, even after extensive aging (3a), the adhesion strength decreases by only 12.8%, which is significantly lower than the rate of decline in the undoped films. This indicates that La-N co-doping significantly improves the mechanical stability of the thin films. Combined with SEM observations, the decline in mechanical properties is primarily associated with the expansion of surface pores, the extension of cracks, and the weakening of grain boundaries. Nevertheless, the doped films maintain strong substrate–film adhesion throughout the UV aging process, further validating the enhancing effect of La-N co-doping on interfacial bonding and effectively mitigating the deterioration of mechanical properties during aging. Overall, the increase in adhesion strength from 256 μN (undoped) to 936 μN (co-doped), combined with only a 12.8% degradation over 3 years of UV aging, underscores the critical role of La-N co-doping in enhancing long-term mechanical reliability.

Furthermore, although UV aging was performed up to an equivalent of 5 years, the main characterization and analysis focused on the first 3 years, since the film properties began to deteriorate significantly after this point. Extending the study beyond 3 years was constrained by practical considerations and the diminishing stability of the films, making the 0–3 year period most relevant for evaluating aging resistance.

## 4. Conclusions

This study systematically analyzed the effects of different durations of UV aging (0, 1, 2, and 3 years) on the structure and properties of La-N-TiO_2_ thin films. Employing techniques such as XRD, SEM, AFM, Raman spectroscopy, contact angle testing, and nano-scratch testing, the investigation focused on the phase structure, surface morphology, hydrophobicity, and mechanical properties of the thin films, leading to the following conclusions:

### 4.1. Structural Stability

XRD analysis indicates that La-N co-doping significantly suppresses the transformation from the anatase to the rutile phase during UV aging, thereby maintaining the crystalline stability of the thin films. SEM and AFM results demonstrate that La-N co-doping enhances the density of the films, inhibits grain growth and crack propagation, and results in only a 62% increase in surface roughness after three years of UV aging, displaying exceptional resistance to UV-induced aging.

### 4.2. Mechanical Properties

Results from nano-scratch tests reveal that La-N co-doping significantly strengthens the interfacial bonding, effectively mitigating the deterioration of the mechanical properties of the thin films during UV aging. Even after three years of UV exposure, the films maintain commendable wear resistance and adhesion, exhibiting excellent mechanical stability.

### 4.3. Water Resistance

Contact angle measurements show that La-N co-doping substantially enhances the hydrophobicity of the thin films. Even after three years of UV aging, the contact angle remains at 82°, far superior to that of pure TiO_2_ films. This indicates that La-N co-doping effectively delays the decline in hydrophobic properties of the films, providing strong support for their long-term resistance to hydrolysis and weathering.

### 4.4. Practical Implications and Future Outlook

The enhanced structural, mechanical, and hydrophobic stability of La-N co-doped TiO_2_ thin films under prolonged UV exposure suggests strong potential for real-world applications, particularly in self-cleaning coatings for photovoltaic modules, architectural glass, and outdoor sensor surfaces. These materials are especially suitable for regions with high solar irradiance and dust pollution, where long-term performance is essential.

However, this study primarily focused on UV-induced aging under controlled conditions. Environmental factors such as rain erosion, temperature cycling, acid/base corrosion, and airborne particulates were not included, which may also affect material performance. Therefore, further studies are necessary to assess long-term durability under multi-factor environmental stressors.

Future research will also explore alternative co-doping strategies, evaluate the synergy of La-N doping with anti-fouling or anti-reflection properties, and carry out real-world outdoor exposure tests to verify practical feasibility and long-term reliability.

## Figures and Tables

**Table 1:** UV aging cycle.

Aging Time/Years	Irradiation Duration/Hours	Simulated Time/Days
0 years	0 h	0 days
1 year	48 h	2 days
2 years	96 h	4 days
3 years	144 h	6 days
5 years	240 h	10 days

**Table 2:** AFM scans data.

UV Aging Time	Average Grain Diameter (nm)	Average Roughness (pm)	Root Mean Square Roughness (pm)
0 years	2.462	422.6	541.9
1 year	2.893	623.4	801.7
2 years	3.199	648.5	818.8
3 years	4.288	683.3	865.0

## Data Availability

The original contributions presented in this study are included in the article. Further inquiries can be directed to the corresponding author.

## References

[1] Soni N., Singh P.K., Mallick S., Pandey Y., Tiwari S., Mishra A., Tiwari A. (2024). Advancing Sustainable Energy: Exploring New Frontiers and Opportunities in the Green Transition. Adv. Sustain. Syst..

[2] Dong W., Ma M. (2025). Recent developments and advanced applications of promising functional nanocomposites for green buildings: A review. J. Build. Eng..

[3] Yakubu S., Samikannu R., Gawusu S., Wetajega S.D., Okai V., Shaibu A.-K.S., Workneh G.A. (2025). A holistic review of the effects of dust buildup on solar photovoltaic panel efficiency. Sol. Compass.

[4] Bora B., Rai S., Dhar A. (2023). Effect of UV irradiation on PV modules and their simulation in newly designed site-specific accelerated ageing tests. Sol. Energy.

[5] Abdullah M., Hosain M.M., Parvez M.M.H., Motayed M.S.H. (2025). Prospects and challenges of thin film coating materials and their applications. Inorg. Chem. Commun..

[6] Wang Y.-H., Rahman K.H., Wu C.-C., Chen K.-C. (2020). A Review on the Pathways of the Improved Structural Characteristics and Photocatalytic Performance of Titanium Dioxide (TiO_2_) Thin Films Fabricated by the Magnetron-Sputtering Technique. Catalysts.

[7] Dai L., Fu P., Chen J., Sun F. (2023). Nitrogen doping mediated oxygen vacancy and Ti valence regulation to enhance photocatalytic H_2_ generation. Int. J. Hydrogen Energy.

[8] Chu J., Sun Y., Han X., Zhang B., Du Y., Song B., Xu P. (2019). Mixed titanium oxide strategy for enhanced photocatalytic hydrogen evolution. ACS Appl. Mater. Interfaces.

[9] Vijayarangan R., Bharathkumar S., Mohan S., Valdes H., Ilangovan R., Amin M.A., Vyas S., El-Bahy Z.M. (2024). Rationalizing Fe-Modified TiO_2_ through doping, composite formation, and single-phase structuring for enhanced photocatalysis via inter- and intra-charge transfers. Mater. Sci. Eng. B.

[10] Wan B., Liu X., Qi L. (2024). Research progress of TiO_2_-based photocatalytic CO_2_ reduction. Chin. J. Appl. Chem..

[11] Yang Z., Ma Z., Ren J., Xiong Y., Ren F. (2025). Theoretical and experimental studies on the optical properties of La-doped TiO_2_ with oxygen vacancies. Opt. Mater..

[12] Zhu X., Pei L., Zhu R., Jiao Y., Tang R., Feng W. (2018). Preparation and characterization of Sn/La co-doped TiO_2_ nanomaterials and their phase transformation and photocatalytic activity. Sci. Rep..

[13] Chakraborty A.K., Ganguli S., Sabur M.A. (2023). Nitrogen doped titanium dioxide (N-TiO_2_): Electronic band structure, visible light harvesting and photocatalytic applications. J. Water Process Eng..

[14] Xu J., Wu Y., Zhang S. (2019). Effects of rare earth element doping on the structure and photocatalytic performance of TiO_2_ thin films. J. Ceram..

[15] Xiong J., Liu Y., Xia L., Jiang G., Xiao D., Mishra Y.K. (2025). Electrochromic properties of cobalt-doped titanium dioxide films. Ionics.

[16] Guner B., Safikhani-Mahmoudi M., Li F., Zou K., Dagdeviren O.E. (2025). Ultraviolet irradiation penetration depth on TiO_2_. Commun. Chem..

[17] Zhao H., Wu H., Shi B., Wang J., Wu C., Wang C., Wang X., Liu W., Dai C., Wang D. (2025). Fast and controllable anatase-to-rutile phase transition irradiated by NIR light. Chin. Chem. Lett..

[18] Pan Y., Bai T., Tian Y. (2013). Effects of UV Irradiation on the Optical Properties of TiO_2_ Films. J. Appl. Opt..

[19] Luo X., Wang J., Wang C., Zhu S., Li Z., Tang X., Wu M. (2016). Degradation and mineralization of benzohydroxamic acid by synthesized mesoporous La/TiO_2_. Int. J. Environ. Res. Public Health.

[20] Shen Z., Li Y., Ding Y. (2015). Ground Simulation Methods for the Effects of UV Radiation on Aerospace Materials. Spacecr. Environ. Eng..

[21] (2013). Plastics—Methods of Exposure to Laboratory Light Sources—Part 2: Xenon-Arc Lamps.

[22] Vittadini A., Casarin M., Selloni A. (2006). Chemistry of and on TiO_2_-anatase surfaces by DFT calculations: A partial review. Theor. Chem. Acc..

[23] Li H., Wan J., Dang W.H. (2011). Preparation and Characterization of Nano-TiO_2_/PE Antibacterial Films. Adv. Mater. Res..

[24] Singh K., Harish S., Hayakawa Y., Shimomura M. (2023). Experimental and theoretical study of oxygen vacancy induced La-doped mesoporous TiO_2_ for enhanced thermal stability. AIP Adv..

[25] Sikam P., Thirayatorn R., Kaewmaraya T., Thongbai P., Moontragoon P., Ikonic Z. (2022). Improved Thermoelectric Properties of SrTiO3 via (La, Dy and N) Co-Doping: DFT Approach. Molecules.

[26] Sun L., Zhao X., Cheng X., Sun H., Li Y., Li P., Fan W. (2012). Synergistic Effects in La/N Codoped TiO_2_ Anatase (101) Surface Correlated with Enhanced Visible-Light Photocatalytic Activity. Langmuir.

[27] Qu M., Huang G., Liu X., Nie X., Qi C., Wang H., Hu J., Fang H., Gao Y., Liu W.-T. (2022). Room temperature bilayer water structures on a rutile TiO_2_(110) surface: Hydrophobic or hydrophilic?. Chem. Sci..

[28] Huang Y., Cao J.-J., Kang F., You S.-J., Chang C.-W., Wang Y.-F. (2017). High Selectivity of Visible-Light-Driven La-doped TiO_2_ Photocatalysts for NO Removal. Aerosol Air Qual. Res..

